# Radiographic Evaluation of Nano-Propolis, Nano-Curcumin and MTA as Direct Pulp Capping Agents in Young Permanent Teeth

**DOI:** 10.4317/jced.62497

**Published:** 2025-09-01

**Authors:** Eman M Hassan, Dalia M Elasser, Dina M Abdel-Ghany, Shereen Fathy, Sabah M Sobhy

**Affiliations:** 1 Lecturer of oral medicine, periodontology oral diagnosis and radiology Department, Faculty Of dental Medicine for Girls, Al-Azhar University, Cairo, Egypt; 2 Lecturer of Operative Dentistry Department, Faculty of Dental Medicine for girls, Al-Azhar University, Cairo, Egypt; 3 Lecturer of Paediatric Dentistry and Dental Public Health Department, Faculty of Dentistry, October 6 University; 4 Lecturer of oral and maxillofacial radiology, Faculty of Dentistry, October 6 University, Giza, Egypt; 5 Endodontic Department, Faculty of Dental Medicine for Girls, Al-Azhar University, Cairo, Egypt

## Abstract

**Background:**

To assess the ability of nano propolis and nano curcumin as direct pulp capping agents in forming dentin bridge in permanent teeth using the CBCT.

**Material and Methods:**

For the present investigation, fifty-four teeth were selected and randomly divided into three groups (18 teeth per group) based on the pulp capping agent utilized: Group I (control group): teeth were directly pulp capped with MTA. Group II: they were directly pulp capped with propolis nanoparticles. Group III: they were directly pulp capped with curcumin nanoparticles. Cone-beam computed tomography (CBCT) scans were performed on all teeth following pulp capping therapy and six months later to assess the establishment of reparative dentin bridges. In order to detect reparative dentin bridges on the multi-planar reconstruction picture, the CBCT scan was examined. A statistical analysis was conducted.

**Results:**

reparative dentin bridges were formed in all teeth across all patient groups. Group III (Nano-curcumin) had the greatest mean value, followed by group II (Nano-propolis), while Group I (MTA) had the lowest value.

**Conclusions:**

When applied as a direct pulp capping agent, Nano curcumin and Nano propolis induce the production of reparative dentin and apex closure in permanent teeth.

** Key words:**MTA, Nano-propolis, nano-curcumin, young permanent teeth, direct pulp capping, CBCT.

## Introduction

The primary benefit of vital pulp therapy is preservation and maintenance of the pulpal tissues that have been damaged by deep caries, trauma, previous restorative procedures or iatrogenic reasons. Vital pulp therapy has been approached in a variety of strategies including indirect or direct pulp capping and pulpotomy [1,2]. Direct pulp capping is a process in which a small pulpal exposure is capped with a protective material that forms a microbiological tight seal. This approach can be used when pulpal exposure occurs via uninfected dentine during caries excavation or crown preparation. It is indicated to perform direct pulp capping during the same session in which the exposure occurs either during mechanical cavity preparation or immediately following trauma. Direct pulp capping should only be carried out on a vital tooth that shows no history of spontaneous pain, apical pathology, mobility or any signs of pulpal necrosis at the site of exposure [3,4].

Most direct pulp capping materials cause superficial necrosis after placement directly over the exposed pulp tissue. Due to the existence of this necrosis, the vital pulp cells that lie beneath it are protected from the alkaline pH of the material. Additionally, it enables the underlying pulp cells to perform the repair and regeneration processes [5]. During reparative dentinogenesis, the original odontoblasts at the exposure site are destroyed and stem/progenitor cells are migrated to the injured site, followed by their subsequent proliferation and differentiation into odontoblast-like cells. Reparative dentinogenesis is often initiated by the deposition of a fibrodentin matrix, which is atubular and irregular and is associated with cuboidal shaped cells. Subsequently elongated and polarized odontoblast-like cells produce a more organized tubular dentin like matrix [6]. The criteria for success include asymptomatic tooth showing no sensitivity to percussion, palpation, or pressure, and no spontaneous pain. Additionally, there should beno sinus tract, swelling or tooth mobility. Radiographically, there should be no evidence of radiolucency in the furcation area or periapical region [7,8]. A variety of materials been suggested for direct pulp capping of the exposed pulp. Calcium hydroxide has become one of the most popular and traditional materials for pulp capping in permanent dentition due to its antimicrobial properties which lead healing and repair of the mineralized tissues [9] but it has some limitations such as lack of sealing ability, pulp surface inflammation and necrosis and tunnel defect in the newly formed dentin bridge [10] These restrictions have been removed with the usage of mineral trioxide aggregate (MTA). Tetra calcium alumino ferrite, tricalcium silicate, dicalcium silicate, tricalcium aluminate, and gypsum. It has low solubility, high biocompatibility and a good sealing and reparative dentin-forming ability [11]. Previous clinical trials have shown excellent success rate ranging from 90% to 100% for direct pulp capping procedures using MTA, after 1 year of follow-up [12-14]. However, it has some disadvantages, including long setting time, high cost and difficulty in handling [15] With the evolution of nanotechnology in dentistry, several nanomaterials have been developed as promising alternatives for the traditional materials. Nanomaterials have sTable structures, improved mechanical properties, decreased crack propagation, improved bonding to the tooth structure and excellent antibacterial properties [16]. Metallic nanoparticles have been shown to have good antimicrobial effect but they may have un favorable side effects. Consequently, there is a switch in the research to use natural nanoparticle products among these are nano proplis and nano curcumin [17].

Propolis is a resinous substance that honey bees collect from different plant species. The most important pharmacologically active constituents in propolis are flavonoids, phenolics and aromatics. Propolis exhibits a wide range of biologic activities, including antimicrobial, antioxidant, immunomodulatory and anti-inflammatory properties that encouraged using it in dentistry for many treatments as caries prevention, direct and indirect pulp capping, root canal disinfection, a storage media for avulsed teeth and accelerating surgical wound healing [18]. It has been shown that propolis is a more efficient pulp capping agent compared to calcium hydroxide. It inhibits microbial infection, inflammatory process and pulpal necrosis and stimulates reparative dentin formation [19].

Another natural material is Curcumin (CUR); The ginger family, which originated across half of Asia, includes Curcuma longa as a major bioactive polyphenol component. This substance displays antioxidant, antimicrobial and anti-inflammatory properties [20]. When comparing the effects of curcumin (CUR) and Nano-curcumin (N-CUR) on mesenchymal stem cells (MSCs) derived from systemically healthy individuals with chronic periodontitis, N-CUR demonstrated an increase in cell survival rates, even after a 72-hour incubation period [21]. One of the most prominent signs of successful direct pulp capping, as observed in radiographs, is the presence of an opaque bridge [22]. Even though non-invasive methods for evaluating mineralized reparative dentin, such as periapical radiography and parallel long cone radiography, are frequently used in daily practice, they are unable to detect the earliest signs of the formation, making it impossible to compare studies [23]. Thus, it becomes feasible and accurate to evaluate hard tissue in three dimensions [24]. The position of the reparative dentin is supported by the CBCT scan, which may also measure the targeted item qualitatively and quantitatively [25].

The purpose of this study was to evaluate dentin bridge formation and apex closure when nano propolis and nano curcumin were used as direct pulp capping agents in young permanent teeth by using CBCT. The radiographic evaluation of nano-natural materials (nano propolis and nano curcumin) as direct pulp capping agents in young permanent teeth does not differ from that of MTA, according to the null hypothesis.

## Material and Methods

Materials.

Materials used in the current study, their description, manufacturer, and composition are found in ( Table 1).

Methods:

1. Propolis nanoparticles Preparation:

After putting 100 g of raw propolis in a flask with 500 mL of 80% ethanol, the mixture was filtered through filter paper and let to sit on a warm plate with a stirrer (MSH-20A, Wetige®, Germany) for seven days. To isolate the pure propolis particles, distilled water was added to the purified solution at a 1:10 ratio. To remove the proplis nanoparticles, the suspension was then submerged in an ultrasonic bath for 20 to 30 minutes. The process yielded nano propolis in a colloidal form [26]. (Nano Gate com., Nasr City, Cairo, Egypt).

2. Curcumin nanoparticles preparation:

For Curcumin loaded zinc oxide nanoparticles 7.5%, weight 0.925gm of nano ZnO powder dispersed in 50ml ethanol with stirring and sonication for 30 min then 0.075 gm of native curcumin were added and left-over night with stirring in closed vessel after that, the composite was dried at 60 oC for 24h [26]. (Nano Gate com., Nasr City, Cairo, Egypt).

3. Clinical Study.

3.1 Trial design and setting:

This study was designed as interventional, randomized, prospective clinical trial and was conducted in the clinic of pediatric Department and Dental Public Heath, Faculty of Dental Medicine.

3.2 Sample size calculation:

Sample size calculation was performed using G*Power version 3.1.9.2, Faul *et al*. [27]. University Kiel, Germany. The effect size f was 0.44 (large) according to the Vu TT *et al*. [28]. with alpha (α) level of 0.05 and Beta (β) level of 0.05, i.e., power = 80%; the estimated sample size (n) should be 54 samples and will be divided equally into 3 groups (18 samples/group) 

3.3 Eligibility criteria’s:

Fifty-four patients were selected for the present study from patients that were referred to the clinic of pediatric Department and Dental Public Heath after clinical and radiographic examinations according to some criteria. The patients who met the inclusion criteria to be of age category between 7 and 13 years, noncontributory medical condition and complaining of permanent molars – where with mature or immature apices- showing signs of reversible pulpitis with pinpoint pulpal exposure not exceeding 2 mm. Cases with pulpal exposure due to deep carious lesion, trauma, previous restorative procedures or iatrogenic causes were included.

The exclusion criteria were patients with irreversible pulpitis, uncontrolled bleeding of the pulp (more than 10 minutes) after its exposure, pulp necrosis, tenderness to percussion, abnormal tooth mobility, swelling or the presence of a sinus tract. Teeth that were non-restorable tooth and radiographic signs of external or internal resorption, calcified canals, inter-radicular bone loss or periapical pathology were also excluded.

3.4 Trial registration and ethical approval:

This clinical trial was registered online in ClinicalTrials.gov. All data regarding the aim of the study, number of patients enrolled, inclusion and exclusion criteria, primary outcome and starts and estimated trial completion dates were provided. The registry identification number was NCT06029023 / Date: January 30, 2024.

With code number RECO6U/8-2023, the study was authorized by the Faculty of Dental Medicine’s Research Ethics Committee (REC).

3.5 Informed consent:

All patients’ parents signed the informed consent form after being informed with the treatment benefits and risks, number of visits and expected duration of the research. All informed consent forms were written in Arabic language to be easily understood by the patients and their parents.

3.6 Sample grouping, randomization and blinding:

For this study, fifty-four teeth in total were chosen, and they were split into three groups at random (18 teeth each) based on the pulp capping agent that was used.

Group I (control group): teeth were directly pulp capped with MTA.

Group II: they were directly pulp capped with propolis nanoparticles.

Group III: they were directly pulp capped with curcumin nanoparticles

An independent physician used Excel software to create a random sequence in order to verify randomization prior to the current investigation. After the pulp capping process was finished and before the material was applied, the operator was notified of the random sequence, which was stored in opaque, sealed envelopes.

**Figure 1:**
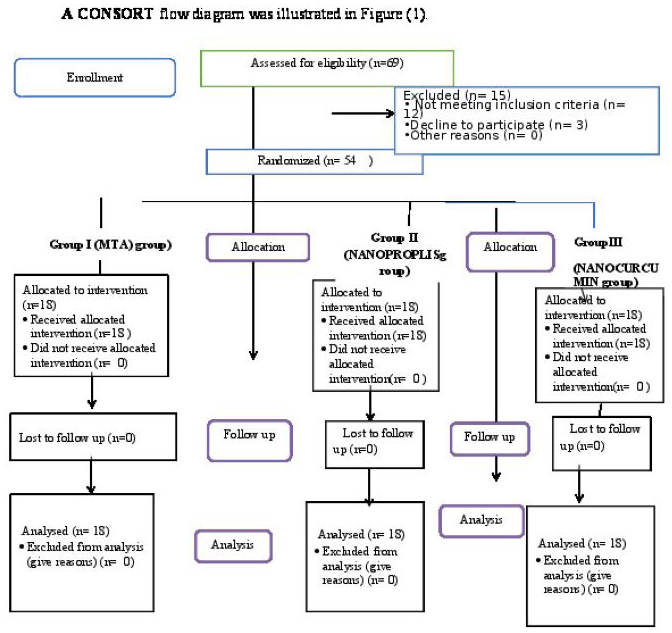
A CONSORT flow diagram was designed in the present study.

The outcome assessor and data analyzer in the current study took double blinding into consideration, (Fig. 1).

4. Treatment protocol:

Following the administration of rubber dam isolation and local anesthetic (4% articaine with 1:100,000 epinephrine), a spoon excavator was used to remove the carious soft dentin near the pulp, and a high-speed round bur with irrigation was used to prepare the cavity contour. After pulp exposure was verified and the pulp tissue satisfied the requirements for inclusion, 2.5% sodium hypochlorite (NaOCl) was used to disinfect it. Hemostasis was achieved by pressure over the exposed pulp using cotton pellets moistened with 2.5% NaOCl for 10 minutes. After this procedure Persistent hemorrhagic pulps (> 10 min), the tooth was excluded from the study. saline solution was used for the final rinse to remove any remaining sodium hypochlorite, followed by drying the cavity with a cotton pellet.

 Subsequently, the teeth were randomly assigned to one of the three experimental groups

Group I (MTA group):

The manufacturer’s instructions for mixing white MTA (ProRoot; Dentsply maillefer) were followed, using a 1:3 water-to-powder ratio. The exposed pulp and surrounding dentin were then covered with MTA at a thickness of 1.5 mm using an amalgam carrier. Appropriate adaptation and condensation techniques were employed to ensure complete coverage of the exposure site, facilitating effective sealing and tissue response. A moistened cotton pellet was placed over MTA, and the tooth was temporarily restored using intermediate restorative material (IRM, Dentsply Tulsa Dental). After 24 h, the cavity was lined with glass ionomer cement.

Group II (Propolis nanoparticles group) and Group III (Curcumin nano particles group):

In the nanopropolis and nanocurcumin groups, propolis was injected from a pre-loaded syringe onto a sterile glass slab, then transported to the exposure site with a metal carrier. Collagen sponge (Guangdong Victory Biotech, China) was trimmed to dimensions of 2.5 × 2.5mm and was placed as a barrier over the applied material. To ensure optimal application of nano propolis and nano curcumin, the consistency of the mixture should be carefully observed before placement. The nanoparticle solution should be well-dispersed and homogenous to maximize bio availability and therapeutic efficacy. The use of sterile glass slab provides a smooth surface for even distribution, preventing clumping and ensuring a controlled amount of material is applied. Additionally, the collagen sponge serves as a mechanical barrier, preventing premature material loss and maintaining localized effects by creating a stable microenvironment for tissue healing and regeneration. Using a well calibrated metal carrier allows for precise delivery to the exposure site, reducing the risk of contamination or excess material placement.

Final restoration:

For composite resin placement as a final restoration, all cavity walls were firstly itched with phosphoric acid 37% followed by the application of All Bond Bisco universal adhesive (BISCO, Inc. Schaumburg, U.S.A) according to manufacturer instructions. Composite layers were eventually applied and light cured incrementally at 20_second each. Finishing and polishing were performed. Patient were subsequently scheduled for follow-up appointment.

Follow up:

Preoperative and follow-up CBCT were performed at base line and 6 months. Thickness of formed dentin bridge were assessed using CBCT. Evaluation was performed by two calibrated examiners.

Radiographic evaluation:

Utilizing the ProMax® 3DX-ray devices (Planmeca, Helsinki, Finland), digital CBCT images were obtained. The parameters of 90 kVp, 14 mA, and 0.1 mm3 voxel size were used to create CBCT exposures. The diameter and height of the field of view were 5 cmand5cm, respectively. In order to detect reparative dentin bridges on the multi-planar reconstruction picture, the CBCT scan was examined. Romaxis software is used to measure the thickness of dentine bridges. Three perpendicular lines—the mesial pulp horn, the distal pulp horn, and the middle of the pulp chamber—were drawn from the line that connected the tooth’s mesial and distal cemento-enamel junctions to the base of the restoration. to enable the estimation of the reparative dentin bridge’s volume [29].

Statistical analysis:

The values were displayed using the mean and standard deviation (SD). The data was examined for normality using Shapiro-Wilk test. The results of the normality test indicated that the parametric data had a normal distribution. As a result, the ANOVA test was used for intergroup comparisons, and the Bonferroni post hoc test was used for pairwise comparisons. The paired t test was used to compare baseline and follow-up results within the same group. The qualitative data (root closure) was represented by counts and percentages. Chi square was used for all intragroup and intergroup comparisons. *P*≤0.05 is considered significant. Statistical analysis was performed using the computer program SPSS software for windows version 26.0 

## Results

I-Inter-group Comparison (Between Groups at Baseline and Follow-up): ( Table 2).

At Baseline: There was a highly significant difference between the groups at baseline (F test= 114.47; *p-value* <0.001). Post-hoc analysis (indicated by superscript letters) shows that Group III (Nano curcumin) had a significantly higher baseline value (2.24±0.10) compared to Group I (MTA) and Group II (Nano propolis), which were not significantly different from each other (1.32 for both).

At Follow-up: There was also a highly significant difference between the groups at follow-up (F test= 51.19; *p-value* <0.001). Post-hoc analysis indicates that Group II (Nano propolis) and Group III (Nano curcumin) had significantly higher follow-up values (2.56 and 2.78 respectively) compared to Group I (MTA) (1.70). Interestingly, Group II and Group III were not significantly different from each other at follow-up.

Intra-group Comparison (Baseline vs. Follow-up): All three groups (Group I - MTA, Group II - Nano propolis, and Group III - Nano curcumin) showed a highly significant increase in the measured parameter from baseline to follow-up, as indicated by the paired t-test *p-value*s of <0.001 for each group. This suggests that all treatments were effective in increasing the parameter within their respective groups.

Root closure: (Table 3)

The Table presents both intra-group and inter-group comparisons of root closure within the same group, focusing on the Chi-square test results. Inter-group analysis at baseline shows significant differences between the three groups, with Group I (MTA) and Group II (Nano propolis) having 100% open roots and no closed roots, while Group III (Nano curcumin) has a lower percentage of open roots (66.7%) and some closed roots (33.3%), with a *P* value of 0.003 indicating significant differences. At follow-up, the trend persists with significant inter-group differences (*P* = 0.003), suggesting that the groups differ notably in root closure outcomes.

 In intra-group comparisons, both Group I and Group II show highly significant changes (*P* < 0.001**), with roots shifting from open to closed status after treatment, indicating effective root closure within these groups. Conversely, Group III shows a non-significant change (*P* = 0.068 ns), implying that the treatment did not produce a statistically significant effect on root closure within this group. Overall, the intra-group analysis suggests that Group I and Group II experienced significant root closure post-treatment, while Group III’s improvement was not statistically significant despite some increased closed roots, and the inter-group analysis highlights the significant differences in root closure outcomes among the different treatment groups.

## Discussion

Pulp capping is a minimally invasive surgery that seeks to reduce bacterial contamination, stimulate reparative dentin development, and preserve pulpal vitality, which is especially important in teeth with incompletely formed roots. This procedure’s success rate is determined by a variety of parameters, including age, gender, pulp exposure site, tooth type, and pulp capping material [2]. A variety of materials have been proposed for directly capping the exposed pulp. The application of a medicament directly to the exposed pulp tissue is essential for promoting pulp healing and producing reparative dentin. If effective, this approach will eliminate the need for more invasive, extensive, and costly treatment [30].

Vital pulp therapy has accelerated due to biocompatible materials with regenerating potential. MTA’s widely recognized biocompatibility, superior biological seal, and tissue regeneration capabilities have made it the most widely used material. When compared to traditional pulpotomy medications, MTA has shown significant success rates in various studies as a pulpotomy drug in primary molars [6,7,9,10]. As a result, in the current investigation, we used MTA as the control group [31]. Natural therapies from traditional medicine have been used in dental procedures to overcome MTA limits such as handling, expense, and setup time.

The bee gathers propolis, a resinous material that is said to offer numerous health benefits for people. This content has been researched in a number of dental specialties [32]. Nano Propolis reduces inflammation by inhibiting the lipoxygenase pathway and producing TGF-β1, which promotes the creation of dentine bridges by promoting odontoblast differentiation and collagen synthesis [26]. Nano curcumin was tested in this study due to its anti-inflammatory activities which is comparable to MTA. Nano curcumin is a highly pleiotropic substance that interacts with a number of inflammation-related biological targets. Curcumin has demonstrated potent anti-inflammatory, antioxidant, and immunomodulatory properties [30]. The main ways that nano curcumin promotes dentine bridge development in the dental pulp are by its regenerative and anti-inflammatory qualities. It stimulates the mineralization process required for bridge building and allows dental pulp stem cells to differentiate into odontoblasts, the cells that make dentine. Furthermore, by preventing infection of the exposed pulp, its antibacterial qualities aid in the establishment of a favorable environment for dentine bridge creation and healing [21].

The presence of an opaque dentin bridge on the radiograph is one of the most important indicators of the success of the direct pulp capping procedure [33]. Despite being widely used noninvasive techniques for evaluating mineralized reparative dentin, such as periapical radiography and parallel long cone radiography, these methods are unable to identify the earliest signs of the formation and make it impossible to compare studies [23]. Cone beam computed tomography (CBCT) makes it feasible and accurate to evaluate hard tissue in three dimensions. The reparative dentin’s position and both qualitative and quantitative measurements of the target object are made easier by the CBCT scan [24].

By comparing average dentin thickness after six months between different study groups. Our investigation found that there was also a highly significant difference between the groups at follow-up (F test= 51.19; *p-value* <0.001). Post-hoc analysis indicates that Group II (Nano propolis) and Group III (Nano curcumin) had significantly higher follow-up values (2.56 and 2.78 respectively) compared to Group I (MTA) (1.70). Interestingly, Group II and Group III were not significantly different from each other at follow-up. These findings are in line with that of Abuhashema, A. R. *et al*. [29] who found that after six months, there was a statistically significant increase in the remaining carious dentin density in both groups. Furthermore, compared to the control group, the study group’s carious dentin density increased statistically significantly. The study group’s mean tertiary dentin thickness was 0.164± 0.081 mm, while the control groups was 0.148± 0.134 mm.

According to previous research, the antibacterial properties of curcumin, which is a pulp capping agent, can account for the current study’s findings. The region surrounding the 5% nCur-ABBL discs displayed the highest growth inhibition zone [34]. When antimicrobial photodynamic therapy (aPDT) was coupled with a gradual increase in nCur concentration from 0.5% to 5%, the number of *Streptococcus mutans* colonies within the biofilm decreased statistically substantially over time (*P* < 0.05). Additionally, nCur-ABBL discs containing 2% and 5% nCur shown similar antibacterial activity at all evaluated time points up to 60 days (*P* > 0.05). Aghazadeh *et al*. [32], in contrast to our investigation, reported that, at a 9-month follow-up, teeth treated with MTA exhibited more appropriate clinical and radiographic outcomes in comparison to propolis. The current study showed that apex closure after six months all cases of group I (MTA) and group II (Nanopropolis) had closed apex, while in group III (Nanocurcumin), 66.7% of cases had closed apex and 33.3% had open apex. The difference between group III and the other two groups was statistically significant (*p*=0.003).

Results according to Damle S. G. *et al*. [35] are consistent with this investigation, which showed that after 9 months, barrier development was shown in 90.90% of patients in the MTA group and 81.81% in the Ca(OH) group. In the Ca(OH) group, the average barrier formation time was 5.33 months, whereas in the MTA group, it was 4.90 months. Because of MTA’s high alkalinity (pH of 12.5), the results were explained. MTA’s higher pH promotes alkaline phosphatase activation and boosts antibacterial action. Furthermore, calcium-dependent pyrophosphatase activity is stimulated by high calcium concentrations which facilitates lesion asepsis and starts the bone healing [33]. This study supports the potential use of bioactive nanomaterials derived from natural sources in vital pulp therapy, though longer-term investigations are warranted.

Limitations of this study include the small sample size, potential bias due to the absence of long-term follow-up, and the exclusion of irreversible pulpitis cases, which may limit generalist ability. Furthermore, long-term outcomes such as pulp regeneration or failure rates require further investigation.

## Conclusions

Both nanocurcumin and nano propolis have been shown to promote reparative dentin formation and apex closure in permanent teeth when applied as direct pulp capping agents.

## Figures and Tables

**Table 1:** Materials used in this study and their manufactures.

Material	Manufacturer
Mineral trioxide aggregate (MTA)	ProRoot; Dentsply maillefer
Nano propolis	Nano Gate com., Nasr City, Cairo, Egypt
Nano curcumin	Nano Gate com., Nasr City, Cairo, Egypt
Glass ionomer	Vitrebond, 3M ESPE, St. Paul, MN, USA
Composite resin	Filtex Z350, 3M ESPE, Salt Lake City, UT, USA

**Table 2:** Descriptive statistics and comparison of dentine thickness, in different groups (ANOVA test).

	Baseline		Follow up		Paired T-test	P value
	Mean	SD	mean	SD		
Group I (MTA)	1.32 b	0.16	1.70b	0.17	-5.563	<0.001**
Group II (Nano propolis)	1.32b	0.23	2.56a	0.27	-9.305	<0.001**
Group III (Nano curcumin)	2.24a	0.10	2.78a	0.36	-6.525	<0.001**
F test	114.47		51.19			
P value	<0.001**		<0.001**			

Significance level *p*≤0.05, *significant Post hoc test: means sharing the same superscript letter are not significantly different

**Table 3:** Descriptive statistics and comparison of root closure between and within the same group (Chi square test).

	Group I (MTA)		Group II (Nano propolis)		Group III (Nano curcumin)		P value between groups
	Open	Closed	Open	Closed	Open	Closed	
Baseline	15 (100%)	0	15 (100%)	0	10 (66.7%)	5 (33.3%)	0.003*
Follow-up	0	15 (100%)	0	15 (100%)	5 (33.3%)	10 (66.7%)	0.003*
P value with the same group	<0.001**		<0.001**		0.068 ns		

Significance level *p*≤0.05, *significant, ns=non-significant

## Data Availability

The datasets used and/or analyzed during the current study are available from the corresponding author.
